# The Effectiveness of Psychological Intervention for Women Who Committed Child Sexual Abuse: An Empty Systematic Review

**DOI:** 10.1177/15248380241277274

**Published:** 2024-09-17

**Authors:** Marta Sousa, Cláudia Gouveia, Bárbara Freitas, Sónia Caridade, Olga Cunha

**Affiliations:** 1 Psychology Research Center, University of Minho, Braga, Portugal; 2 Digital Human-Environment Interaction Lab, Universidade Lusófona Do Porto, Portugal

**Keywords:** treatment, women who committed child sexual abuse, intervention effectiveness, systematic review

## Abstract

The topic of sex offenses committed by women has received relatively little attention until recent decades. The scarcity of research in this domain underscores the urgent need for a more comprehensive understanding and effective interventions. Women who committed child sexual abuse (CSA) exhibit a distinct psychological profile compared to men, requiring specific intervention programs. The current systematic review has two aims: the primary aim is to analyze the effectiveness of psychological interventions with women who committed CSA, and the secondary aim is to identify the intervention models and strategies used with them. Six databases were searched for studies examining the effectiveness of psychological interventions. All retrieved papers were excluded after full-text assessments as none met the primary objective. However, eight publications fulfilled the secondary objective of identifying models and strategies that could inform intervention development. The intervention programs presented addressed specific targets focused on criminogenic needs highlighted in the literature as central to this population, as well as targets that complement the intervention according to individual characteristics. Moreover, the studies frequently emphasize an intervention approach centered on individuals’ potential (and not just their deficits), employing a gender paradigm to underpin psychological interventions with this population. The results of this review highlight a major gap in the current literature concerning both the delivery and effectiveness assessment of psychological intervention for women who committed CSA. The studies incorporated for the second objective elucidated critical treatment targets and models to inform intervention strategies, which are subsequently discussed in terms of their practical implications.

Child sexual abuse (CSA) is considered a major public health problem within research and politics. The high prevalence rates and devastating outcomes for victims, their families, and society make CSA a worrying phenomenon (Barth et al., 2013; Hailes et al., 2019). According to data from the World Health Organization (WHO, 2022), approximately 1 in 5 women and 1 in 13 men disclose experiencing sexual abuse before the age of 17 years prevalence of CSA has been disparate when assessed using self-reported and official data, with self-reporting methods showing higher rates (Stoltenborgh et al., 2011). A recent meta-analytical review found that only about 2% of cases are officially reported. In contrast, victimization surveys indicate that the prevalence rates of women who committed CSA are six times higher than those suggested by official data (Cortoni et al., 2017). Therefore, many abusive acts go unreported due to some factors, including fear, shame, and a lack of support and understanding from others (Lemaigre et al., 2017; Tozdan et al., 2019). Specifically, victims often perceive the support they will receive after reporting the crime negatively (Lemaigre et al., 2017; Tozdan et al., 2019). In fact, healthcare or justice system professionals often respond inappropriately to these cases based on their stereotypes (Tozdan et al., 2019).

Stereotypically, CSA implies the image of a male who committed a sex crime against a female child. As research in this field has expanded since the 1980s (Cortoni, 2015), it has been established that individuals of any gender, including women, can commit to CSA (Tozdan et al., 2019). In the past, minimal attention was given to traditional gender roles portraying women as nurturing, protective, non-aggressive, and non-sexual traits thought to render them incapable of harming a child (Denov, 2004; Jennings, 1994). As a consequence, early theories on female sexual offending frequently contextualized it either within the framework of male coercion or severe mental health problems (i.e., psychological disorders or personality disorders) (Cortoni, 2015). Currently, limited resources are also allocated to studies on women who committed CSA, reflecting the relatively small number of occurrences perpetrated by women (Gerke et al., 2020). However, women who committed CSA encompass a heterogeneous forensic population with distinct treatment requirements (Hales & Gannon, 2022). Besides, the evolution of contemporary knowledge on women who committed CSA and women who committed crimes in general highlights the inadequacy of applying theories and empirical knowledge developed for males (Gannon et al., 2008). Moreover, there was a lack of risk assessment tools for women who committed CSA, which can complicate accurate risk assessment (e.g., Cortoni, 2010). Risk assessment tools developed for men may overestimate the risk of sexual recidivism in women and fail to identify the unique treatment needs of women, which are essential for informing appropriate treatment plans (Cortoni, 2010).

Psychological and clinical literature has highlighted key differences between men and women who committed CSA (Williams & Bierie, 2015). Concerning offense patterns, women who committed CSA victimized more adolescents and were significantly less likely to be intoxicated at the time of the crime than men (Williams & Bierie, 2015). The same applies to psychological variables, where women who committed CSA show a high level of sexual victimization, and a high prevalence of mental health problems (e.g., personality disorders, substance use disorders, and mood disorders) (e.g., Augarde & Rydon-Grange, 2022; Christopher et al., 2007; Comartin et al., 2018; Gillespie et al., 2015; Miller & Marshall, 2019). Although they share some similarities with men who committed CSA, the motivations for the offense include female-exclusive motives, which are related to personal and/or situational factors (i.e., offending to please a man and being pressured or manipulated by a male partner) (Brown & Kloess, 2020).

Prepared with this knowledge, researchers began to determine whether women who committed CSA could be categorized into specific subgroups or typologies (Mathews et al., 1989). Nevertheless, the available typologies are limited (Hales & Gannon, 2022). Specifically, the typologies are often derived from studies with small samples and selection biases (Bickley & Beech, 2001). Until very recently, there have been no theoretical frameworks within common typologies that could comprehensively elucidate the various interacting factors contributing to women engaging in sexual offenses (Blake & Gannon, 2018). Then, Gannon et al. (Gannon et al., 2008, 2010, 2012) developed the Descriptive Model of Female Sexual Offending. This model aims to explain how and why certain women committed CSA, including cognitive, affective, and behavioral factors. As a result of these model, three pathways were identified: the explicit-approach pathway, that is, women who demonstrated varied motives for their offensive behaviors (e.g., seeking revenge or intimacy), were not selective in choosing their victims and offended against children and adults, effectively self-regulated, and experienced positive emotions linked to their misconduct; the direct-avoidant pathway, that is, women who were unwilling to partake in sexually illegal behaviors with children but were coerced into doing so by a male accomplice, either out of fear or to establish intimacy, experiencing severe negative affect); and the implicit-disorganized pathway, that is, women with different aims for their offensive behaviors, which were generally not premeditated at either distant or immediate time points, and displayed a lack of selectivity in choosing their victims, but offended against either adults or children and experiencing either positive or negative affect (Gannon et al., 2008).

The typologies and the Descriptive Model of Female Sexual Offending have provided some information about the key targets for intervention. Specifically, offense-supportive cognitions, relationship difficulties (e.g., dependency), difficulties in managing emotional regulation and unhelpful coping strategies (e.g., substance use), and deviant or inappropriate sexual interests need to be considered in psychological intervention (Cortoni & Gannon, 2013, 2016; Wijkman et al., 2010). The Descriptive Model of Female Sexual Offending and its subtypes of women who committed CSA highlight that some intervention targets may be a priority for some and not for other women (Gannon et al., 2008). As such, women who committed CSA in the directed avoidant typology may benefit from an intervention focused on improving their relational skills and assertiveness. On the other hand, women listed in the implicit-disorganized approach might find support valuable in enhancing emotional regulation and acquiring effective coping mechanisms. Moreover, considering the characteristics of these women, researchers argue that interventions need to understand more about how women deal with previous abusive experiences and how this is related to the abuse they have perpetrated, focusing first on traumatic experiences (Cortoni, 2018; Williams et al., 2019). Furthermore, interventions should also target mental health disorders and substance addiction (Pflugradt et al., 2018; Williams et al., 2019). However, despite the current empirical attention given to women who committed CSA to understand the risk and maintenance factors contributing to their reduction (Cortoni, 2015), few studies still exist on the criminogenic needs of women who committed CSA, and there are limited intervention programs tailored to this population (Cortoni & Gannon 2013, 2016).

Therefore, the primary aim of the present systematic review was to analyze the psychological interventions with women who committed sex crimes, analyzing their effectiveness. In addition, the secondary aim was to identify the intervention models and strategies used with this population. The identification of these models and strategies may aid in the development of intervention responses.

## Method

The current systematic review was conducted according to Preferred Reporting Items for Systematic Reviews and Meta-Analysis (PRISMA) guidelines (Moher et al., 2010) and registered on OSF REGISTRIES [reference: 10.17605/OSF.IO/4RCVP].

### Search Strategy

The search was conducted in June 2023, without any data restrictions, by two independent researchers with an MSc in Applied Psychology. The following equation search was used to ascertain the pertinent articles: (treatment OR intervention OR therapy OR program* OR psychotherapy OR rehabilitation) AND (“child abuser*” OR “child sexual abuser*” OR child pornograph* OR child molest* OR pedophil* OR paedophil* OR “internet sex* offend*” OR “sexually offend*” OR “sexual offend*” OR “sexual offen*” OR “sexual abuse*”) AND (women OR female). The search was conducted using the following databases: PubMed, ScienceDirect, SCOPUS, EBSCO (PsycInfo), Web of Science, and Scielo. Moreover, the reference lists of systematic reviews and meta-analyses of this population were checked.

### Inclusion and Exclusion Criteria

For the first aim of the current systematic review, the PICOS framework was used to define the inclusion criteria: Participants (P), Interventions (I), Comparisons (C), Outcomes (O), and Study Designs (S) (Thomas et al., 2021). Inclusion criteria were: studies with adult women who perpetrated sexual crimes against children (P), who underwent a psychological rehabilitation program (I), and having or not a comparison group (C). We define psychological rehabilitation as any structured therapeutic activity aimed at addressing psychological, emotional, and behavioral issues in individuals. Moreover, the primary outcome was the effectiveness of psychological intervention, assessed through clinical changes and/or recidivism rates (O). We defined clinical change as the presence of statistically significant differences between baseline and post-test in the dimensions assessed. All types of study designs were considered for this review (S). Exclusion criteria were: (a) literature review; (b) reviews, systematic reviews, and meta-analyses; (c ) studies that collected and analyzed only qualitative data (e.g., interview); (d) papers not peer-reviewed (i.e., gray literature), and (e) studies not written in English, Portuguese, or Spanish. Papers in English, Portuguese, and Spanish are included because all authors are proficient in these languages.

A secondary objective was delineated considering the lack of papers that meet the inclusion criteria during full-text screening. For the second aim, the inclusion criteria were: (a) empirical studies, books, and chapters that describe specific intervention models, strategies, or programs for women who committed CSA; and (b) were written in English, Portuguese, or Spanish. No restrictions regarding the year of publication, or research design were used.

### Literature Selection Process and Data Extraction

Studies identified through equation search were imported into Rayyan software (Ouzzani et al., 2016) to delete duplicates and screen titles and abstracts against the inclusion criteria. Two raters independently screened article titles and abstracts, selecting papers for full-text analysis. Discrepancies between the two raters were resolved through discussion, with a third reviewer available to intervene if needed.

Articles that met the inclusion criteria were retrieved and fully read to reach a final decision by two independent raters. For the first aim of the current systematic review, a codebook was developed to extract data from all included articles, including information about reference details (e.g., authors and year); studies’ characteristics (e.g., location); samples’ characteristics (e.g., size, age, and ethnicity/race); design’s characteristics (e.g., design type and length of follow-up); intervention’s characteristics (e.g., setting, modality, number of sessions or hours, and complementary intervention); measurement’s characteristics (e.g., assessment measures and assessment of recidivism); intervention’s results (e.g., dropout/completion rate and efficacy).

To collect data for the second aim, a new codebook was developed to extract data, including information about references and the intervention’s characteristics (e.g., number of intervention programs, name of the programs, number of sessions, targets of intervention, models of intervention, and description of intervention strategies).

### Quality Assessment

The Quality Assessment Tool for Studies with Diverse Designs (QATSDD) was used (Sirriyeh et al., 2011) to assess the risk of bias in the included studies. We chose to use the QATSDD because it was specifically developed to assess the quality of studies with heterogeneous designs in the field of Psychology and is widely used in systematic reviews. Despite some limitations and the existence of a revised version, the QATSDD has demonstrated strong reliability and validity (Harrison et al., 2021). The QATSDD is a 16-item tool, with two indicators for qualitative studies and two for quantitative studies. The items were scored between 0 and 3, with a higher score indicating greater methodological rigor. Scores on the QATSDD can range from 0 to 42 for qualitative and quantitative studies or up to 48 for mixed-methods studies. To ensure accuracy, the QATSDD was developed to provide an overall quality rating expressed as a percentage.

### Results

**Figure 1.**
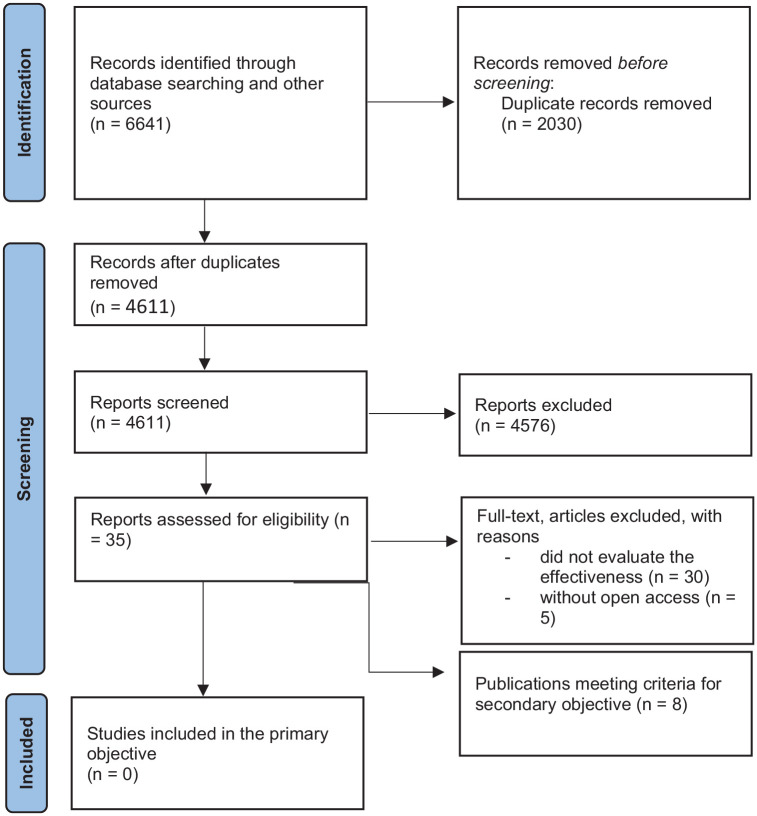
Preferred Reporting Items for Systematic Reviews and Meta-Analysis 2020 flow diagram for new systematic reviews, which included searches of databases, registers, and other sources.

In total, 6,641 articles were retrieved from the data searches and through supplementary searches. After removing duplicates, 4,641 titles and abstracts remained and were screened for relevance. Thirty potentially relevant studies were selected for full-text analysis. Following the full-text assessment, all studies were excluded based on the inclusion criteria. Considering the main objective of this review, no publications were identified that presented the effectiveness of psychological interventions for women who committed CSA (see Figure 1). Studies were excluded because they addressed offenders in general without specifying the type of crimes, did not involve psychological interventions or intervention models but rather other types of interventions, such as pharmacological treatments, or were primarily focused on population characterization.

Since the search yielded no results, a subset of eight publications was identified, focusing on a secondary objective: theoretical discussions of psychological interventions, including strategies that could inform intervention designs. This endeavor adhered to the guidelines outlined in the Cochrane Effective Practice and Organization of Care manual for reporting empty reviews (Yaffe et al., 2012).

### Quality Assessment

The QATSDD provides a percentage score to compare reporting quality across studies. To facilitate article comparisons, three categories were assigned: low quality (≤50%), acceptable quality (51%–74%), and high quality (≥75%) (Crowe et al., 2011). In this systematic review, we included articles of acceptable quality (*n* = 1) and high quality (*n* = 7).

The study by Cortoni and Gannon (2013) had the highest quality score (92%), followed by three studies with an 83% quality score (Blanchette et al., 2010; Costa et al., 2023; Pflugradt & Allen, 2019). Additionally, three studies had a quality score of 75% (Ashfield et al., 2010; Gannon & Rose, 2008; Loinaz, 2016), and one had a score of 67% (Pflugradt et al., 2018).

### Study Results

Four studies presented several intervention programs designed for women who committed CSA (Blanchette et al., 2010; Costa et al., 2023; Gannon & Rose, 2008; Loinaz, 2016), while another four presented treatment models and targets (Ashfield et al., 2010; Cortoni & Gannon, 2013; Pflugradt & Allen, 2019; Pflugradt et al., 2018).

#### Interventions

The four studies included presented nine intervention programs: the programs applied in Lucy Faithfull Foundation (Blanchette et al., 2010; Gannon & Rose, 2008), in the Counseling Institute of Texas (Blanchette et al., 2010), in the Wisdom Works Counseling Services of Colorado (Blanchette et al., 2010), in Warren County Probation (Blanchette et al., 2010), in Saratoga County Probation Department (Blanchette et al., 2010), in Brazil (Costa et al., 2023), the Canadian Sex Offender Therapy Program (Gannon & Rose, 2008), and the Women Sex Offenders Program (Loinaz, 2016). Three programs were conducted in community/probation services, two in community and federal institutions, one in prison, one in a hospital, and one in independent child protection agencies. One study did not report information about it. Only one intervention program was directed at women who committed CSA, with the other focused on women who committed sex crimes in general.

Many of the intervention targets were common throughout the programs described. As such, six interventions focused on cognitive distortions and/or schemas, six on interpersonal issues (such as communication, intimacy, and sexuality problems), six on the offense cycle, five on victim empathy, four on self-regulation, three on relapse prevention plans, and two on sexual arousal (see Table 1).

**Table 1.** Synthesis of Relevant Studies.

	Treatment Program for Female Sexual Offenses	Counseling Institute of Texas	Wisdom Works Counseling Services of Colorado	Warren County Probation	Saratoga County Probation Department	Psychosocial Intervention	Lucy Faithfull Foundation	Canadian Sex Offender Therapy Program	Women Sex Offenders Program
Author (year)	Blanchette et al. (2010)	Blanchette et al. (2010)	Blanchette et al. (2010)	Blanchette et al. (2010)	Blanchette et al. (2010)	Costa et al. (2023)	Gannon and Rose (2008)	Gannon and Rose (2008)	Loinaz (2016)
Population target	Women who perpetrated sex crimes	Women who perpetrated sex crimes	Women who perpetrated sex crimes	Women who perpetrated sex crimes	Women who perpetrated sex crimes	Women who perpetrated sex crimes	Women who perpetrated sex crimes against children	Women who perpetrated sex crimes	Women who perpetrated sex crimes
Modules/Phases/sessions (number)	n/a	n/a	4 phases	n/a	n/a	8 sessions	n/a	4 phases	7 modules; 59 sessions
Targets	Self-management. deviant aversion. cognitive distortions. intimacy; relationships; social functioning; empathy and victim awareness.	Cycle of offense; cognitive distortions; Victim empathy; Life skills; Decision making and relapse prevention.	Pre-abuse cycle, abuse cycle, empathy letters, and relapse prevention.	Relationships, thoughts, and life experiences at the time of offense; cognitive distortions; consent, writing apology letters; self-esteem.	History of sexual offenses and general history of offending.	Relationships, identity, victimization, psychosexual immaturity.	Beliefs, schemas, sexual arousal, self-regulation, interpersonal skills, and human needs in prosocial and positive ways.	Self-management, deviant thoughts and fantasies, sexuality, intimacy and relationships, empathy and victim awareness, cognitive distortions, abuse and trauma, substance abuse, emotional management, parenting, community integration programs, and education and employment.	Context of the crime; beliefs; emotion management; sexuality; communication; relationships and functioning in the community.
Additional targets	n/a	n/a	n/a	n/a	n/a	n/a	prior victimization	n/a	n/a
Models of intervention	CBT, positive psychology, gender-sensitive approach	n/a	CBT	n/a	n/a	Psychodrama and Art Therapy	n/a	n/a	n/a
Setting	Community and federal institutions	Community	n/a	Community	Community	Hospital	Independent child protection agency	Prison	Institutions and community
Country	United States	United States	United States	United States	United States	Brazil	United Kingdom	Canada	Canada

Furthermore, certain programs also exhibit specific characteristics. For instance, the Lucy Faithfull Foundation aims to fulfill human needs through prosocial and constructive means while also addressing prior victimization experiences (Blanchette et al., 2010). Similarly, the Canadian Sex Offender Therapy Program incorporates a modular structure with sequential phases designed to address criminogenic needs while integrating gender-sensitive objectives. This specific program, while addressing targets specific to sexual offenses, also integrates elements from other programs. These include cognitive skills development, addressing abuse and trauma, managing substance abuse, emotional regulation, parenting skills, community integration programs, and education and employment initiatives, alongside community monitoring (Gannon & Rose, 2008). In addition, the intervention program applied in Warren County Probation also addressed self-esteem and consent (Blanchette et al., 2010).

Although the intervention program presented by Costa et al. (2023) has the same aims as the other interventions—strengthen protective factors and reduce risk factors to minimize recidivism-, the intervention is different, so it will be explained separately. The paper presents a proposal for a brief group psychosocial care designed for adult women who committed CSA, with eight sessions of three hours each. This psychosocial intervention is guided by predefined themes and incorporates psychodrama and art therapy resources. Themes include awareness of being in a relationship with others; exploration of the concept of identity, recognition of one’s own lifelong victimization and corresponding suffering, recognition of submission present in relationships with other people, especially intimate partners, and reflection on psychosexual immaturity. Additionally, it also focuses on providing information on legislation (Costa et al., 2023).

#### Models of Intervention and Characteristics of Women Who Committed CSA

The studies highlighted some models of treatment, with special attention to models based on positive psychology and gender, namely an intervention approach centered on potential using a gender paradigm (Ashfield et al., 2010; Cortoni & Gannon, 2013; pose that treatment should & Allen, 2019; Pflugradt et al., 2018). On one hand, the strengths-based model facilitates a collaborative process, enabling individuals to identify their skills and resources. This individualized approach assists people who perpetrate crimes in recognizing and developing their potential within relevant social and relational contexts, such as the community or institution (Pflugradt et al., 2018). Within this approach, the authors highlight two models: the Good Lives Model (GLM) developed by Ward and Brown (2004) and Ward and Mann (2004); and the Strengths-Centered Program created by Marshall et al. (2011) (Ashfield et al., 2010; Pflugradt & Allen, 2019; Pflugradt et al., 2018). These models should also be included taking into account a gender-focused approach.

The gender perspective emphasizes the inclusion of integrated mental health and substance abuse treatment services. This includes a strengths-centered treatment model that fosters self-confidence, women-only groups (not mixed in terms of gender), gender-responsive screening, and corresponding assessment tools, along with appropriate and individualized treatment planning (Pflugradt et al., 2018). This approach recognizes that women may require more social connections than men (Pflugradt & Allen, 2019). The authors pointed out that these results are consistent with research indicating that women derive greater benefits from social relationships and support. Consequently, treatment interventions based on relational models have been shown to be effective, enhancing improvements in women’s overall functioning and stress management.

To a lesser extent, the authors also point out that the Risk-Need-Responsivity model is also important for women who committed CSA (Ashfield et al., 2010; Pflugradt & Allen, 2019). In addition, they highlight the role of the therapeutic alliance. Gender sensitivity ensures that the person who committed crimes feels heard, respected, and supported. Establishing emotional and collaborative bonds, introducing the possibility of change, and setting realistic shared goals are key strategies. Furthermore, trauma-focused theory and Miller’s relational theory are emphasized. Trauma-focused theory helps understand recurring relationship and family dynamics themes (Ashfield et al., 2010).

The studies also identify specific targets for intervention, including developing and maintaining skills such as empathy, moral development, and fostering positive relationships. Cortoni and Gannon (2013), as cited in Pflugradt et al.’s (2018) paper, propose that treatment should address five key areas: cognitive processes, emotional processes, intimacy and relationship issues, sexual dynamics, and psychosocial functioning. Similarly, Covington and Bloom (2006) emphasize the importance of creating a therapeutic environment, implementing diverse interventions, addressing practical needs, fostering the development of educational and professional skills, and providing parental education. Moreover, Cortoni and Gannon (2003) highlight the significance of considering women’s cognitive, personality, and learning styles, as well as additional responsiveness factors such as the presence of anxiety or low self-esteem, past victimization experiences, and general psychological distress. These factors can significantly influence their ability to benefit from treatment and should therefore be considered.

## Discussion

While more attention has been directed toward women who committed CSA in recent decades, there is less information available compared to men who perpetrate CSA (e.g., Cortoni & Gannon 2013, 2016). Additionally, limited knowledge exists regarding psychological interventions for women who committed CSA and their effectiveness. Thus, the current systematic review aimed to analyze the effectiveness of psychological interventions for women who committed CSA. Moreover, it sought to identify the intervention models and strategies used with this population as a secondary aim.

The initial planned review found no studies on psychological interventions with women who committed CSA that evaluated their effectiveness. Searches across multiple databases yielded 4,611 publications, which underwent double screening resulting in 35 publications that did not meet the criteria. Although the empirical literature on characteristics and typologies of women who committed CSA has grown in recent years (Cortoni et al., 2017; McLeod, 2015; Williams & Bierie, 2015), these results suggest a lack of research investment directed toward psychological interventions for women who committed CSA. At the same time, these results are also consistent with the lack of empirically validated risk assessment instruments for sexual violence, which are essential for guiding intervention design (Cortoni, 2010). These findings are concerning for several reasons. First, they may suggest that interventions aimed at men have been mistakenly applied to women as well. However, these groups have different characteristics and, consequently, different intervention needs, such as mental health problems (i.e., personality disorders, substance use disorders, and mood disorders) (e.g., McLeod, 2015; Miller et al., 2009; Williams & Bierie, 2015). Second, it reinforces the absence of empirically validated therapeutic responses, which could result in treatments that, while well-intentioned, might not achieve the desired effect and could potentially harm patients. Consequently, treatment providers working with women who committed CSA lack sufficient information about effective interventions to inform their practice.

The secondary objective review encompassed eight publications relevant to the topic. These publications presented intervention programs tailored for women who committed to CSA. The intervention programs outlined in four studies targeted common needs for treatment, including cognitive distortions and/or schemas, interpersonal issues, offense cycle, victim empathy, self-regulation, and sexual arousal. These areas of focus are consistent with the intervention guidelines presented in typological studies for women who committed CSA (Cortoni & Gannon, 2013, 2016; Wijkman et al., 2010) as well as the guidelines presented in the other studies included in this review on this subject (e.g., Pflugradt et al., 2018). In addition, some programs supplemented the intervention by addressing specific characteristics of women who committed these crimes, such as experiences of victimization and/or substance abuse (e.g., Gannon & Rose, 2008). The literature has indicated that women who committed CSA have higher prevalence rates in these dimensions compared to men, with studies pointing out that a history of prior sexual abuse is associated with the most severe and violent offenses against a child (Cortoni, 2018; Williams et al., 2019). Authors highlighted that adversities in childhood establish a groundwork for various interpersonal issues and ineffective coping mechanisms resulting from enduring relational deficiencies and distorted cognitive frameworks concerning oneself and others (Levenson et al., 2015). Practitioners might mistakenly attribute clinical features like flattened affect, hostility, resistance, or manipulativeness to low motivation for change when in reality, these behaviors could have served as crucial protective factors in navigating trauma. In such cases, trauma-informed approaches can prove highly beneficial in the treatment of women who committed CSA (Levenson et al., 2015; Williams et al., 2019). Indeed, trauma-focused psychotherapies, such as Eye Movement Desensitization and Reprocessing and Cognitive Behavioral Therapies, have been implemented with men who have committed sex crimes and could potentially be adapted for use with women (e.g., Ricci & Clayton, 2017). However, the proposal presented by Costa et al. (2023) introduces a new approach that does not follow the established guidelines for intervention with this population, specifically the use of psychodrama and art therapy. Therefore, conducting an analysis of the effectiveness of this approach through hybrid trials would be extremely important.

Moreover, a potential-focused and gender-sensitive approach was the most targeted models for guiding intervention programs, with the GLM (Ward & Brown, 2004; Ward & Mann, 2004) and Marshall’s potential-focused model (Marshall et al., 2011) emerging as options. The GLM has been proposed for intervention with women who committed crimes, grounded in the premise that “offenders want better lives not simply the promise of less harmful ones” (Ward et al., 2007, p. 106). This approach serves as an alternative to the risk-need-responsivity (RNR) model, which often views individuals as collections of problems or risk factors, potentially exacerbating stigmatization and exclusion (Okotie & Quest, 2013). Moreover, the authors argue that by fixating on deficits, there is a risk of overlooking what is significant to the women themselves, thereby neglecting crucial attributes, skills, and resources that could facilitate the rehabilitation process (Van Damme et al., 2017). To the best of our knowledge, only one recent systematic review has specifically examined the outcomes of interventions consistent with the GLM, with over half of the included studies focusing on individuals who committed sex crimes. It concluded that such interventions were at least as effective as standard programs (Mallion et al., 2020). Specifically, some studies involving individuals who committed sex crimes have indicated that the GLM approach has a positive, future-oriented impact (Harkins et al., 2012).

Future studies should consider these guidelines to build intervention programs, being aware, however, that they lack empirical data on their effectiveness in terms of reducing recidivism. It would, therefore, be important to return studies that test its efficacy and effectiveness. To establish the impact of an intervention under optimal conditions, efficacy trials are often the first step. Although these trials might exaggerate the intervention’s success in real-world clinical or health-related environments, they are crucial for confirming whether an intervention has any effect at all. The results from these trials can then guide subsequent research focused on implementation, such as more extensive effectiveness trials. Particularly relevant are hybrid trials, which concurrently assess the intervention’s effectiveness and the outcomes of its implementation. However, evaluating intervention programs with this population can be a difficult task. In the first place, a clinical trial presents numerous ethical and practical problems since it demands that participants be randomly assigned to a treatment group or to a control group without a therapeutic approach. However, a clinical trial with a waiting list control group could be an option to examine its effectiveness. Second, although research in this area is gaining interest, there is still a lack of empirically validated instruments for this population that can assist in carrying out psychological assessments and evaluating change between treatments (Pflugradt & Allen, 2019).

Despite the significant conclusions, there are inherent limitations. The clear limitation of this review is evident in its failure to yield any studies that met the inclusion criteria and formally addressed the research question. It is important to note that this result does not necessarily indicate overly narrow inclusion criteria; rather, it suggests that the field of research on this topic remains underexplored. This underscores a significant gap in our understanding of the effectiveness of psychological interventions for women who committed CSA. Moreover, data collection was limited to studies, books, and chapters written in English, Spanish, or Portuguese that were available online, potentially excluding valuable insights from other languages and sources with restricted access.

## Conclusions

The systematic review aimed to address the critical gap in research on the effectiveness of psychological interventions for women who committed CSA and identify intervention models and strategies used with this population. There is a scarcity of data in this field. Although thirty publications were identified through a full-text review, only eight address treatment targets and models related to women who committed a sex crime against a child in this area. None of these studies evaluated the effectiveness of intervention programs in this specific crime for women who committed CSA.

This review significantly contributes to this area of research by highlighting the importance of effective interventions tailored to address the specific and often individualized offending behavior of women who commit CSA, given its potential for recidivism and significant harm to victims and society as a whole. The importance of this review lies in the fact that women who committed CSA are often underreported due to various factors, such as fear, shame, and lack of support, as identified in previous research. Consequently, the true prevalence and impact of CSA committed by women on society may be underestimated. It also underscores the urgent need to rethink and evaluate other innovative intervention approaches/models and to encourage clinical trials that can attest to their effectiveness (see Tables 2 and 3).

**Table 2.** Key Findings of the Systematic Review.

Key Findings
There exists a paucity of data within this domain.
None of these studies assessed the efficacy of intervention programs for women who committed CSA.
This review emphasizes the distinctions and unique characteristics of female criminality, underscoring the necessity for intervention programs tailored to these specifics.
Studies underscored a focus on individuals’ potential, employing a gender-informed paradigm to shape psychological interventions for women who committed CSA.

*Note.* CSA = child sexual abuse.

**Table 3.** Implications for Research, Practice, and Policy.

Implications
Further research is required to evaluate psychological treatments and/or develop novel interventions for women who committed CSA.
Interventions focused on the specific characteristics of this population must be developed, based on the potential-focused and gender-sensitive approach.
Researchers should use recidivism rates and pre- and post-treatment changes to determine treatment effectiveness and identify factors that help women desist from offending.
Governments should support and invest in the treatment of women who have perpetrated child sexual abuse.
More public services and agencies should be provided for women who have perpetrated child sexual abuse.

*Note.* CSA = child sexual abuse.

Overall, this systematic review had implications for practice, policy, and research. First, this systematic review presents some targets and models that can guide the design of psychological interventions, especially for addressing sex crimes committed by women. Practitioners should consider the risk level of participants and gender differences when designing intervention programs, as this helps determine the appropriate treatment dosage and outcomes. Future research should also place greater emphasis on aligning services and supports with individual needs to enhance the practical application of the RNR model and improve reintegration outcomes. Second, the findings of this systematic review highlight the need for the development of randomized controlled trials to test the effectiveness of these targets and models in changing the risk factors and, consequently, the risk of sexual recidivism. Specifically, researchers should use both recidivism rates and pre- and post-treatment changes because it helps to know if treatment reduces recidivism, promotes changes in criminological factors, and what kind of changes lead women to desist from offending. Third, governments should support and invest in ongoing research focused on the characterization and intervention of women who have perpetrated CSA. Consequently, more public services and agencies for women who perpetrated CSA should be provided (see Table 3).
